# Systematic assessment of different wash solvents for the analysis of small molecule metabolites using mass spectrometry imaging

**DOI:** 10.1007/s00216-026-06534-x

**Published:** 2026-05-01

**Authors:** Barbara Matić, Lieby Zborovsky, Min Qiu, Katrin Jana Frank, Kıvanç Görgülü, Nicole Strittmatter

**Affiliations:** 1 Department of Biosciences, School of Natural Sciences, Technical University of Munich, Garching b. Munich, 85748 Germany; 2 Comprehensive Cancer Center München, Institute for Tumor Metabolism, Klinikum Rechts Der Isar, Technical University of Munich, School of Medicine and Health, Munich, Germany; 3 Institute of Metabolism and Cell Death, Helmholtz Zentrum München, Neuherberg, Germany

**Keywords:** MS imaging, Sample preparation, Wash protocols, Ionisation efficiency, Spatial metabolomics, Tumour

## Abstract

Wash protocols are a simple, commonly used approach to enhance the detectability of low-abundant or poorly ionisable compounds in mass spectrometry imaging (MSI). The washing procedures aim to enhance analyte ionisation by removing interfering metabolites that affect the ionisation efficiency and detection of the target metabolites. However, despite the widespread use of wash protocols in MSI, their impact on small molecule metabolites (SMM) has not been systematically evaluated. In this study, 12 different aqueous and organic wash solvents were investigated to assess their impact on the signal intensities of SMMs in tumour tissue using desorption electrospray ionisation mass spectrometry imaging (DESI-MSI). The added wash steps proved to be a promising tool for increasing detection sensitivity for targeted metabolites, with >90% of analytes investigated here showing increased sensitivity following the optimum wash solvent step. While chloroform was found most efficient in removing lipids overall, the most versatile solvent to significantly enhance the detection of polar and semi-polar metabolites, including amino acids, nucleic acid compounds, sugars, and organic acids, was found to be ethyl acetate. In contrast, water-based washes enhanced fatty acids and lipids while removing hydrophilic metabolites. This study emphasises the importance of adjusting pretreatment protocols to the molecular class of interest and provides a targeted guide for increasing ion detection sensitivity across a broad range of metabolites.

**Graphical abstract:**

**Figure:**
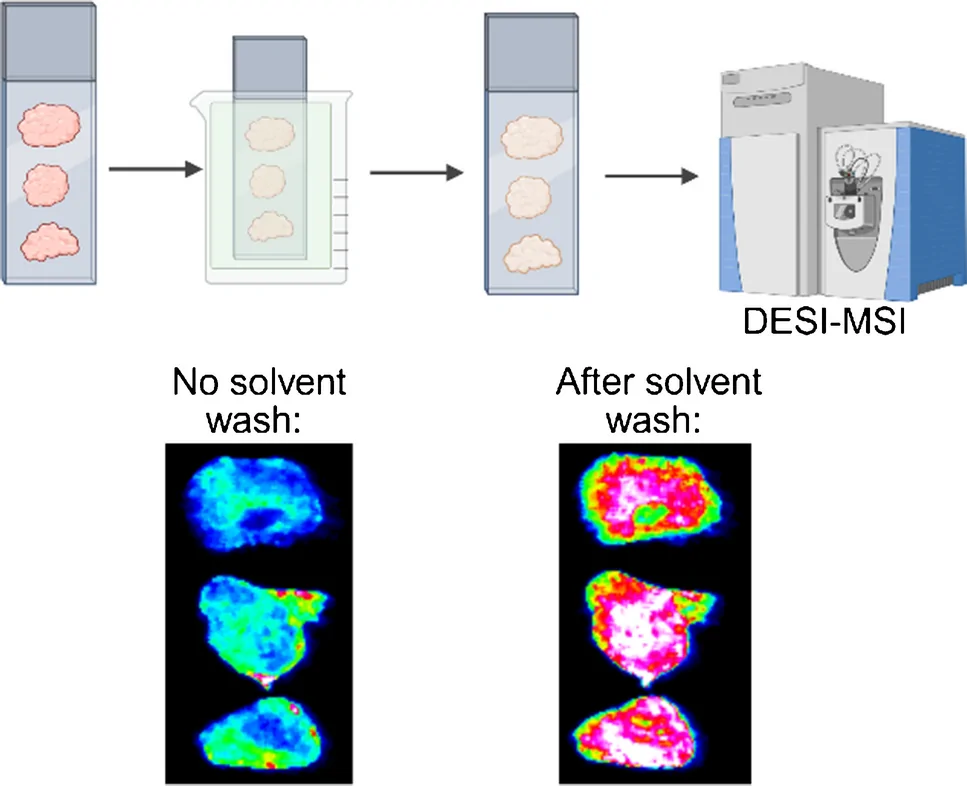

**Supplementary Information:**

The online version contains supplementary material available at https://doi.org/10.1007/s00216-026-06534-x.

## Introduction

Mass spectrometry imaging (MSI) is an increasingly popular technique capable of simultaneously visualising a broad array of endogenous (e.g. metabolites, peptides, lipids) and exogenous molecules (e.g. drugs) in tissue samples in a spatially resolved manner. Although the sensitivity and mass resolving power of MSI instrumentation and methods are continually improving, techniques that enhance the detectability of low-abundant or poorly ionisable compounds in complex matrices remain highly desirable. For specific molecular targets, such as drugs or certain metabolites, selected ion monitoring (SIM) can be used. SIM enables the monitoring of specific mass-to-charge (*m*/*z*) ratios using small mass windows, enhancing the sensitivity of low-abundant compounds [1]. Another method to enhance the detectability of target compounds is on-tissue chemical derivatisation (OTCD) [2]. Here, the target compound is reacted on-tissue with another molecule, resulting in a derivative with improved ionisation qualities. The derivatised target molecule can be visualised either directly or indirectly upon release of the tagging moiety (so-called mass tag approach) [3]. For untargeted lipidomic or metabolic studies, it may be desirable to selectively increase the visibility of specific molecular groups. This can be achieved using ion mobility spectrometry (IMS) coupled with mass spectrometry [4]. In this technique, ions are separated based on their mobility, which is determined by size, shape, and charge, and analysed by MS.

A simpler approach that does not require special equipment is the application of wash protocols. Here, the tissues are washed prior to the MSI measurement using a single or multiple solvent application steps. The washing procedures aim to enhance analyte ionisation by removing molecular groups of lower priority, thereby reducing overall ion suppression for the remaining molecular groups. Therefore, the physico-chemical properties of the applied solvent wash are selected to favour the target molecules. Washing protocols are a prerequisite for on-tissue protein imaging. In different examples, solvent wash steps using alcohols [5–8], organic solvents (e.g. xylene, chloroform [9, 10]), and aqueous buffers [11] were used for the enhancement of protein signals in imaging experiments. In these examples, the enhancement of the protein signals was achieved by washing out lipid molecules, thus minimising signal suppression. To enhance lipid signals, several aqueous buffer washes, such as ammonium formate and ammonium acetate, were found useful [12, 13]. Several examples of the application of solvent washes for imaging small molecule metabolites (SMM) were reported. Various organic solvents, including chloroform, alcohols, acetonitrile (ACN), and acetone, were tested for imaging small metabolites in rat brain tissue using MALDI-MSI [14]. It was reported that a 15-s chloroform wash increased the signal intensities and doubled the number of compounds detected in the *m/z* region of 150–500, while removing a portion of fatty acids and monoacylglycerols [14]. Sun et al. [15] reported that an acetone immersion improved the sensitivity and coverage of MALDI-MS for imaging SMM (e.g. polyamines, cholines, carnitines, amino acids, nucleosides, carbohydrates, organic acids, fatty acids) and easily ionisable lipids, while decreasing the levels of peptides and proteins.

Although wash protocols are routinely used in many MSI-related studies, the selection of suitable washing conditions often relies on personal experience or trial and error. To date, no systematic assessment of the effect of washing conditions on SMM intensity in MSI experiments has been reported.

In this work, we systematically evaluate the effects of 12 commonly applied solvent washes on analyte signal and localisation in DESI-MSI for a variety of metabolite groups, including amino acids, nucleotides, TCA cycle intermediates, fatty acids, and polar lipids. As MSI has become an important tool in basic and applied cancer research, we performed this study on fresh-frozen PANC-1 xenograft tumour tissues. Based on the analysis of this reference dataset, freely accessible on the Metaspace platform [16], we provide a practical guide to help MSI scientists select effective wash treatments tailored to their experimental needs.

## Methods

### Chemicals

Sodium Citrate Buffer pH = 4 with 1% (*w*/*v*) hydroxyethylcellulose, 0.25% (*w*/*v*) Tween, and 0.05% (*w*/*v*) Antifoam A concentrate was purchased from Sigma-Aldrich Chemie GmbH (Taufkirchen, Germany). Acetone, chloroform, dimethyl-sulfoxide (DMSO), methyl tert-butyl ether (MTBE), and a 4% (*w*/*v*) formalin solution in phosphate-buffered saline (PBS) (*v*/*v*) were purchased from VWR International GmbH (Darmstadt, Germany). Ethanol (EtOH), acetonitrile (ACN), and ethyl acetate (EtOAc) were purchased from Sigma-Aldrich Chemie GmbH (Taufkirchen, Germany). Xylene, ammonium acetate salt, and ethylenediaminetetraacetic acid diammonium (EDTA) salt hydrate were purchased from Thermo Fisher GmbH (Kandel, Germany). Phosphate-buffered saline (PBS) tablets were purchased from Th. Geyer GmbH & Co. KG (Renningen, Germany). HPLC-grade water was used to prepare 150 mM EDTA, 150 mM ammonium acetate, and PBS solutions, as well as for additional water washes after PBS, DMSO, and EDTA. HPLC-grade methanol and water, used as spray solvent for the DESI-MSI measurements, were purchased from Sigma-Aldrich Chemie GmbH (Taufkirchen, Germany). The log *K*_OW_ and polarity index of each solvent are provided in Supplementary Table S1.

### Tissue preparation

Tumours were grown by injecting the human pancreatic cancer cell line PANC-1 into the flanks of 6-week-old female nude mice (Crl:NU(NCr)-*Foxn1*^*nu*^, Charles River). Tumours were allowed to grow until an average tumour volume of 150 mm^3^ was reached. To reduce animal numbers according to the 3R principles, these were from a vehicle control cohort and were given 10 µL/g of a vehicle solution containing 50 mM Sodium Citrate Buffer pH = 4 with 1% (*w*/*v*) hydroxyethylcellulose, 0.25% (*w*/*v*) Tween, and 0.05% (*w*/*v*) Antifoam A concentrate daily via oral gavage. Following necropsy, tumour pieces were flash frozen in liquid nitrogen and stored at -80 °C until further analysis. The tumours were then embedded into a single block of 7.5% (*w*/*v*) hydroxypropyl methylcellulose (HPMC) and 2.5% (*w*/*v*) polyvinylpyrrolidone (PVP). The embedded tumours were flash-frozen by submersion in isopropanol at −78 °C, then in 2-methylbutane at −78 °C. The resulting block was kept in a freezer at −80 °C until cryosectioning. The tumour samples were serially sectioned at 10 µm thickness with a Leica CM1950 cryostat (Leica Microsystems GmbH, Wetzlar, Germany) at −20 °C, thaw-mounted onto Superfrost microscope glass slides in rows of three, and dried under nitrogen flow. The sectioned tissues were sent to the Institute of Pathology (Klinikum rechts der Isar, TUM) for hematoxylin and eosin (H&E) staining. The resulting images were examined in ImageScope × 64.

### Wash procedure

The tissues were washed by submerging the glass slide in 40 mL of the solvent for 30 s, except for the 4% (*w*/*v*) formalin wash, which was kept at 5 min to achieve sufficient tissue fixation. On each slide, the middle section remained unwashed (control), while the two outer sections were immersed in different solvents. For the EDTA, PBS, and DMSO washes, an additional 30-s water wash was performed. For ethanol, an additional water submersion was added to rehydrate the tissues.

#### DESI-MSI

Measurements were performed on a home-built automated 2D moving stage equipped with a home-built sprayer assembly [17] mounted to an Orbitrap mass spectrometer (Q-Exactive Plus, Thermo Scientific, Bremen, Germany). Analysis was performed with 3 ppm mass accuracy in negative ion mode using the following instrument parameters: S-Lens RF level of 75, capillary temperature of 320 °C, maximum injection time of 150 ms, and a mass resolution of 70,000 at *m*/*z* 200 in profile mode. The measurements were performed in negative ion mode over a *m/z* range of 80–900, with a spatial resolution of 150 µm. LC–MS grade 95% (*v*/*v*) methanol was used as a spray solvent at a flow rate of 2 µL/min and a spray voltage of ± 2 kV. A Harvard Apparatus Elite syringe pump equipped with a 2.5-mL Hamilton syringe was used for the solvent supply. The solvent was nebulised with nitrogen at a backpressure of 2 bar. All data are deposited on Metaspace; access links are available in Supplementary Table S2.

### Data conversion and analysis

Raw files were converted into.mzML files using ProteoWizard msConvert (version 3.0.4043) [18] and subsequently compiled to an.imzML file using imzML converter version 1.3 [19]. The data was further processed in LipostarMSI 2.1.0 [20] using the following peak detection parameters: a minimum SNR of 0, a noise window size of 0.1 amu, and a minimum absolute intensity of 0.1. Peaks with intensities below 0.2% of the base peak were discarded. Savitzky-Golay smoothing was performed using a window size of 7 points, second order, and 1 iteration. Linear interpolation was preferred for baseline correction with a segment size of 0.1 amu. The dataset was loaded using a 5 ppm *m/z* tolerance, a 1% minimum peak frequency, a 0.1 minimum peak intensity, and a minimum spatial chaos of 0.7. The preprocessing was then followed by manual removal of the remaining images caused by noise.

**Fig. 1:**
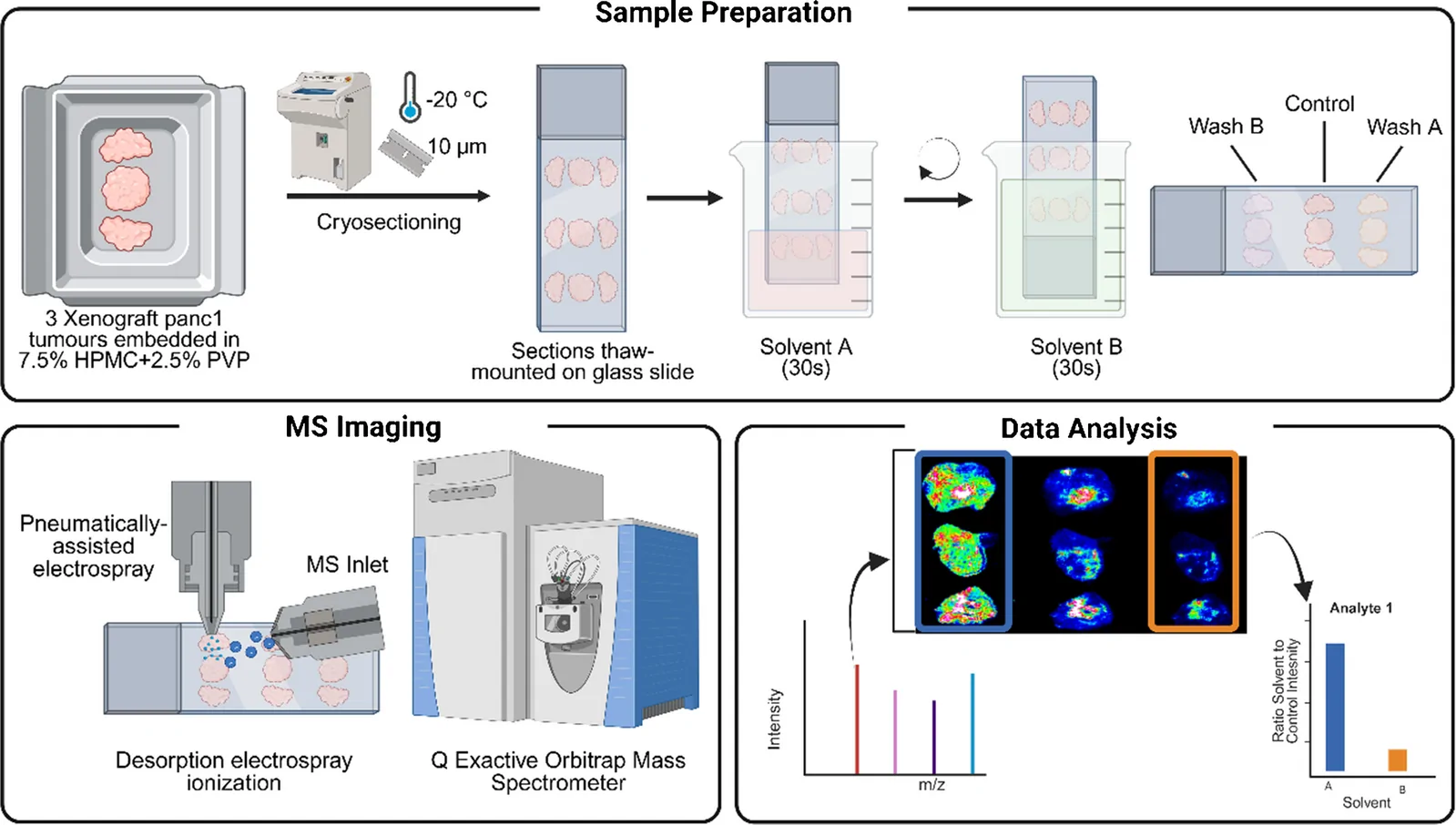
Workflow used in this study. Three PANC-1 xenograft tumours were embedded together and sectioned as a single block. Three consecutive sections were placed on a single microscope glass slide. The middle section served as a control (unwashed), while each of the two outer sections was washed with a different solvent. Following MSI, analyte abundance for each wash solvent was normalised to the control tissue section for the 3 tumour replicates, and the fold-change values for the various metabolites were calculated. Single ion images for several metabolites post-washes are shown in Fig. 2. Created in BioRender. Matic, B. (2026) https://BioRender.com/kgl5pda

Regions of interest (ROIs) were manually drawn around the tumour tissue only following assessment of an adjacent H&E stain to exclude areas of necrosis or muscle tissue (see Supplementary Figure S1). The intensities of the ROIs were denoised (quantile thresholding, hotspot removal = 99.95%) and subsequently processed with a mass tolerance of 5 ppm. All analyses were performed on raw ion intensities to assess signal changes as a measure of detection sensitivity. The area-averaged intensity from a range of known endogenous molecules covering a broad range of polarities was extracted (log *K*_OW_ ranging from −4.9 for glutathione to 13.7 for Cer(34:1)). The full target list, including adduct details as well as FDR thresholds, is provided in Table S3. As sections were consecutive, large changes in tissue composition between control and washed sections were not expected. For tissues on each slide, the average fold-change was calculated in relation to the respective control tissue on a tissue-by-tissue basis (three replicate tumours on each slide, see Fig. 1).

## Results and discussion

In order to provide a community reference dataset and guidelines, we have systematically performed the evaluation of 12 different, commonly used wash solvents following the scheme shown in Fig. 1. The solvents were selected based on previously reported protocols [9–15], covering a range of commonly applied aqueous (PBS, ammonium acetate, EDTA, 4% (*w*/*v*) formalin), and organic solvents (70% (*v*/*v*) EtOH, DMSO, EtOAc, ACN, MTBE, acetone, xylene, chloroform) with a polarity index range from 7.2 (for DMSO) to 2.5 (for xylene and MTBE). The polarity indexes and log *K*_OW_ of all selected solvents are summarised in Table S1.

We assessed the results for (i) the increase of sensitivity for specific molecular groups, ranging from primary metabolites to lipids, and (ii) metabolite delocalisation. While (i) was performed by calculating fold-change ratios between corresponding washed and control tissue sections on the same slide on a biological triplicate of tumour tissues, (ii) was assessed by comparing on-tissue signal intensity to that occurring outside the same tissue. Although the deployed tumour tissue is relatively homogeneous, necrotic tissue areas in tumour replicates #1 and #3, and remaining muscle tissue in tumour replicate #2, were identified and excluded from the ROIs subsequently used for analysis. More information on the metabolites and the solvents assessed can be found in Supplementary Tables S1 and S3.

A wash duration of 30 s was chosen for all tested solvents (except 4% (*w*/*v*) formalin), as this is the typical pre-wash duration used in imaging protocols [5, 14, 21]. A 4% (*w*/*v*) formalin solution is normally applied in tissue imaging experiments as a fixation step. Here, a washing time of 5 min or more is required to achieve sufficient fixation.

**Fig. 2:**
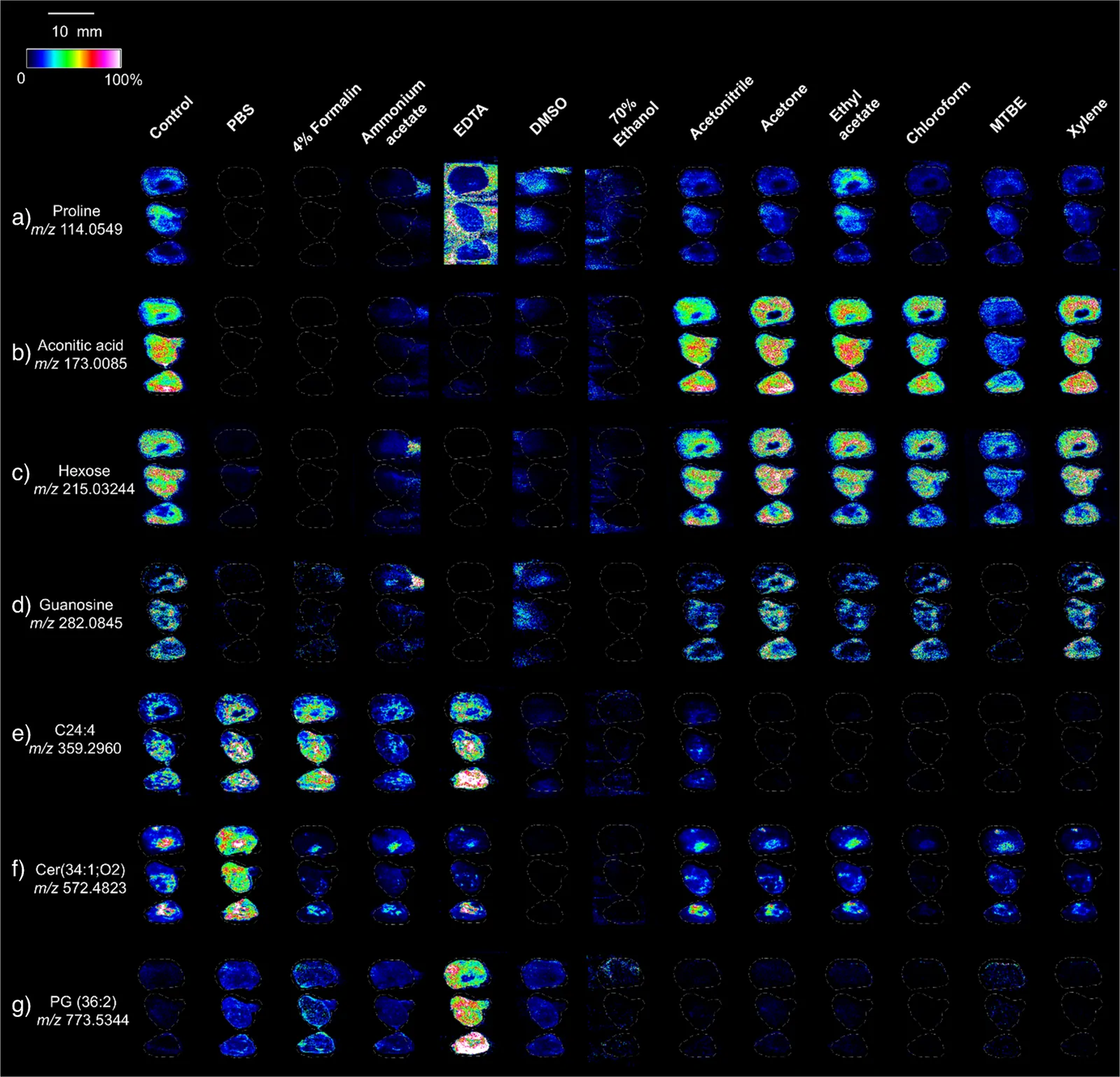
Representative DESI-MS images (± 5 ppm) of various metabolites after washes with different solvents. (**a**) Amino acid: proline *m/z* 114.0459. (**b**) Organic acid: aconitic acid *m/z* 173.0085. (**c**) Sugar: hexose *m/z* 215.0324. (**d**) Nucleic acid compound: guanosine *m/z* 282.0845. (**e**) Fatty acid: C24:4 *m/z* 359.2960. (**f**) Ceramide: Cer(34:1;O2) *m/z* 572.4823. (**g**) Lipid: PG (36:2) *m/z* 773.5344. Spatial resolution: 150 µm

**Fig. 3:**
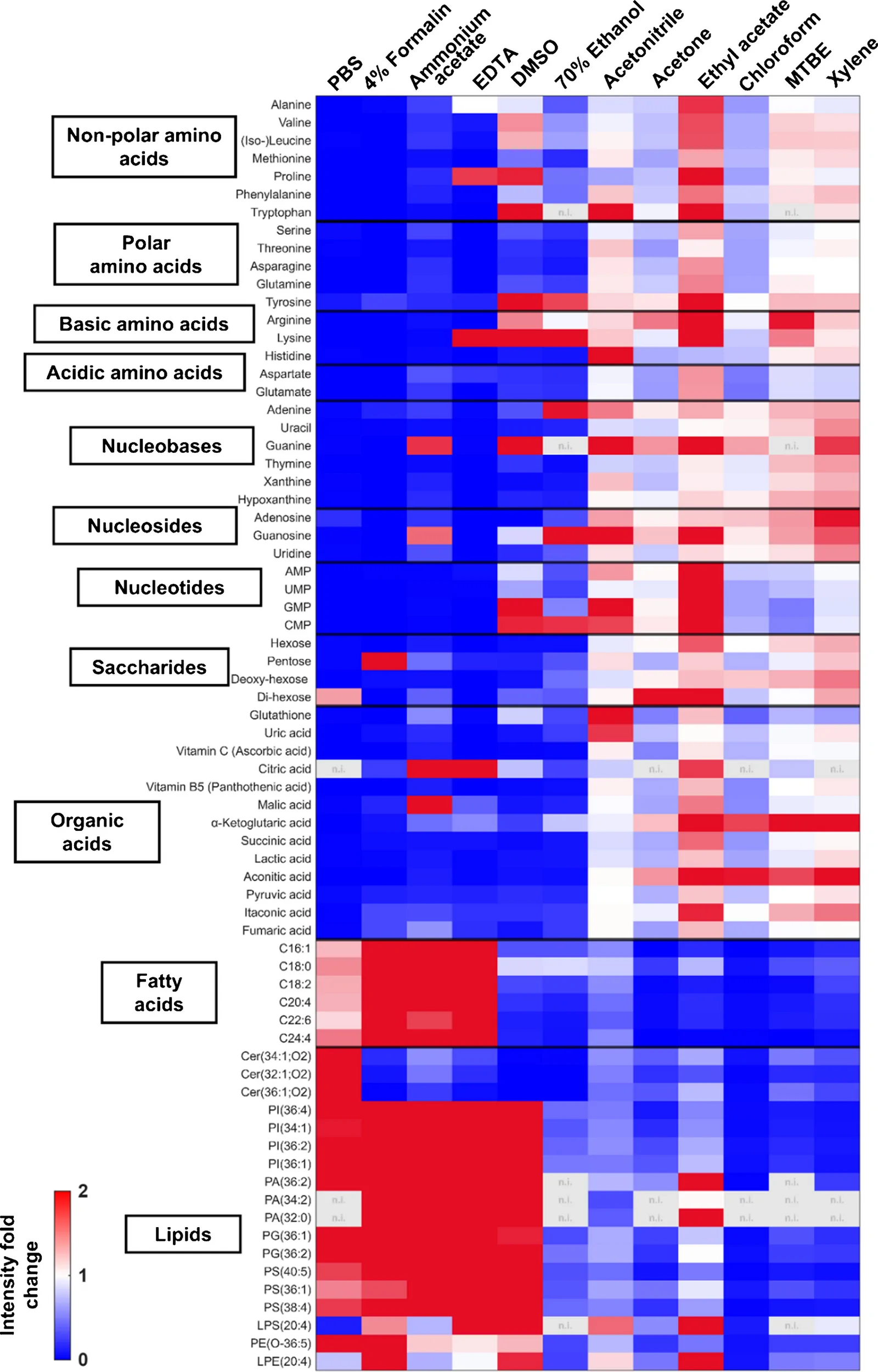
Average intensity fold change in treated vs. control tumours across metabolite classes. Solvents are arranged in order of decreasing polarity (left to right). 0 = complete removal, 1 = no change, ≥ 2 = ≥ two-fold increase. n.i., not detected. Individual tissue data are shown in Supplementary Figure S2

MS images obtained for a selection of metabolites analysed in this study are shown in Fig. 2. Each solvent was compared with a consecutive section control, and fold changes were calculated to assess changes in the average intensity of the respective metabolite. The results of this analysis for tumour tissue are shown in Fig. 3.

The effect of wash solvents on metabolite abundance varied substantially across compound classes and was strongly influenced by solvent polarity. Solvents preferentially dissolve and remove from the tissue surface such compounds with similar physico-chemical properties. Metabolites of opposite polarity are therefore retained within the tissue, allowing them to be ionised more efficiently by reducing signal suppression from competing ions. Consequently, and in line with previous reports, non-polar solvents remove lipophilic metabolites, enhancing the ionisation of polar metabolites, while polar water-based solvents remove hydrophilic metabolites, thereby increasing the ionisation of non-polar metabolites. For each metabolite tested, a wash solvent could be found that increased its signal intensity. The range of enhancement varied from 1.2- to 5-fold for SMM, whereas lipophilic metabolites showed higher enhancements of 2- to 20-fold. However, the quality of solvents varied across metabolite classes (and even within more heterogeneous metabolite classes) and individual metabolites. EtOAc was shown to be most effective for hydrophilic SMM, while EDTA and ammonium acetate increased lipophilic metabolites to the highest degree. Ceramides were uniquely increased with PBS. These distinct patterns emerging for polar and non-polar metabolites are discussed on the basis of compound class in the following section.

### Amino acids

EtOAc was the most effective wash solvent for enhancing the ion intensity of all amino acids, with the single exception of histidine. Most other organic solvents (DMSO, ACN, MTBE, xylene) produced smaller increases, whereas chloroform and acetone generally reduced amino acid signals. Aqueous solvents, PBS, 4% (*w*/*v*) formalin, ammonium acetate, and EDTA, led to a decrease in intensity for almost all amino acids. Responses within subclasses were broadly consistent with this trend, although ACN selectively enhanced larger non-polar amino acids to a higher degree than EtOAc. Lysine showed strong intensity increases but exhibited metabolite delocalisation after EDTA, DMSO, and ethanol washes. A detailed discussion on different amino acid subclasses can be found in the Supplementary Information.

### Nucleic acid components

Organic solvent washes increased the intensity of nucleobases and nucleosides, with the most consistent enhancements observed with EtOAc, xylene, and MTBE. Monophosphorylated nucleotides showed more heterogeneous effects. While acetone and xylene showed little effect on intensity, chloroform and MTBE reduced intensity. EtOAc produced the most significant intensity increases for all detected nucleotides, while ACN provided moderate enhancement. Aqueous washes consistently removed most nucleic acid components, whereas ammonium acetate selectively increased guanine and guanosine but caused delocalisation. Mixed results were observed for DMSO and 70% (*v*/*v*) ethanol.

### Saccharides

Intensity gains through solvent washes in the group of mono- and di-saccharides are generally modest with all solvents tested. The best results were observed with xylene and EtOAc. Other non-polar or moderately polar solvents either preserved analyte intensity or caused slight removal. Although formalin selectively increased pentose intensity, water-based washes removed significant amounts of sugar, consistent with their hydrophilic nature.

### Organic acids

For small organic acids, water-based washes removed most compounds, while EtOAc consistently increased intensities. Other organic solvents showed varying effects across different organic acids. MTBE and xylene predominantly preserved or slightly increased intensities, whereas chloroform reduced signal intensity for most acids.

### Lipids

Fatty acids and lipids exhibited higher intensities following aqueous washes. In contrast, organic solvent washes resulted in consistent fatty acid and phospholipid removal, with increasingly non-polar solvents causing more pronounced decreases in intensity. Chloroform proved most effective for removing lipophilic metabolites. Ceramides were selectively enhanced following PBS washing, while all other polar and non-polar solvents removed this class of lipids. Lysophospholipids were not enhanced as consistently by aqueous washes nor decreased as consistently by a subset of the organic solvent washes.

### Delocalisation

**Fig. 4:**
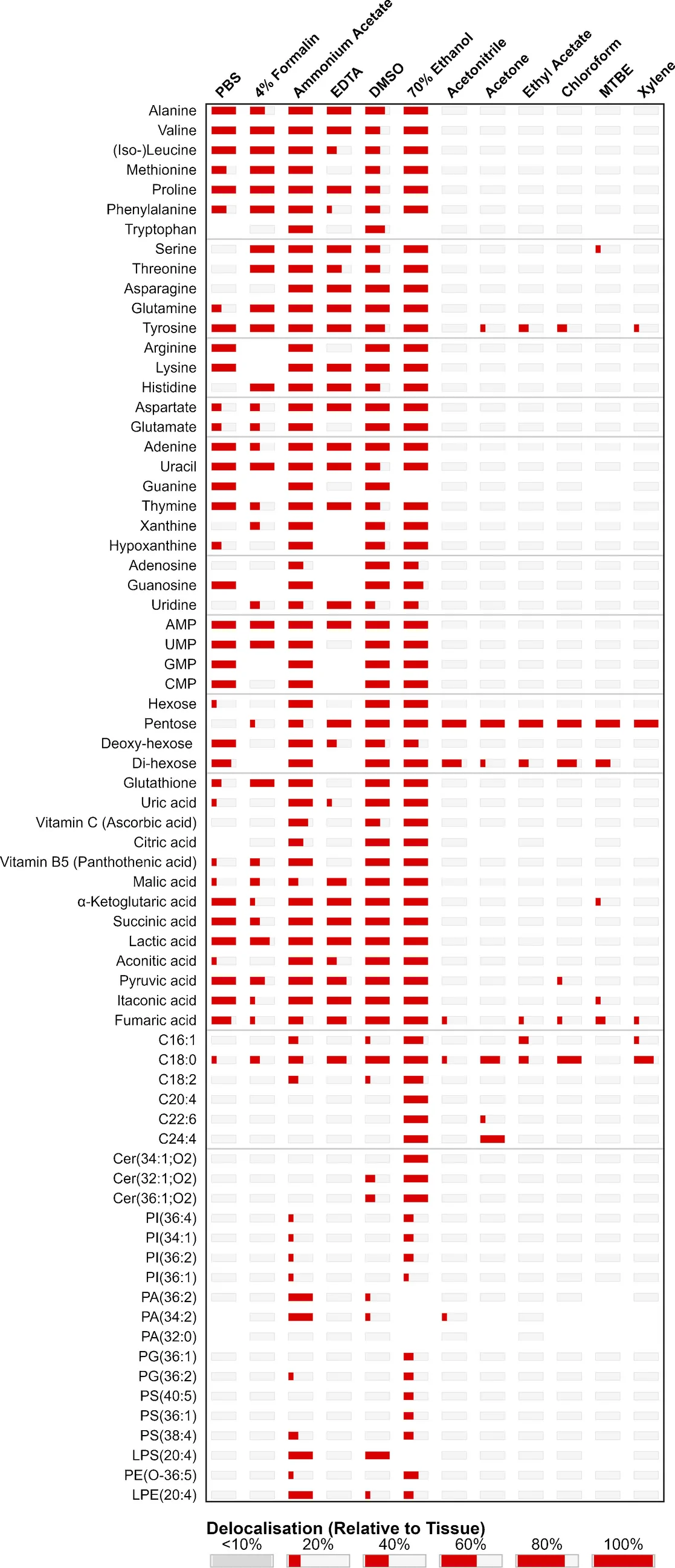
Degree of molecular delocalisation after solvent washing. Red bar icons indicate the percentage of metabolite signal detected outside the tissue relative to the signal in the tumour tissue (intensity outside/intensity within tumour × 100). No bars denote < 10% delocalisation; increasing bars represent thresholds of 20, 40, 60, 80, and 100%

Delocalisation was quantified by calculating the percentage of area-averaged signal intensities surrounding the washed tissue regions at the corresponding *m*/*z* values, relative to the area-averaged intensities in the respective tumour tissue. The resulting degrees of delocalisation are presented in Fig. 4. Varying degrees of delocalisation were observed with water-based and organic solvents, with the latter being more effective at retaining metabolites in tissues. The highest degree of delocalisation was observed after a 70% (*v*/*v*) ethanol wash, with almost all metabolites showing high signals on the glass background. Here, the metabolite signal extended up to 2 mm from the tissue border (Figure S3). Also, the aqueous washes (e.g. ammonium acetate) caused a significant delocalisation of hydrophilic metabolites onto the glass slide surrounding the tissue, indicating incomplete removal. In contrast, less delocalisation of polar metabolites was detected after organic washes, with ACN, EtOAc, and MTBE showing the least delocalisation, comparable with that of the non-treated (control) tissues (Figure S3). Lipophilic analytes were selectively removed, leaving no signal on the glass background.

### Tissue integrity

**Fig. 5:**
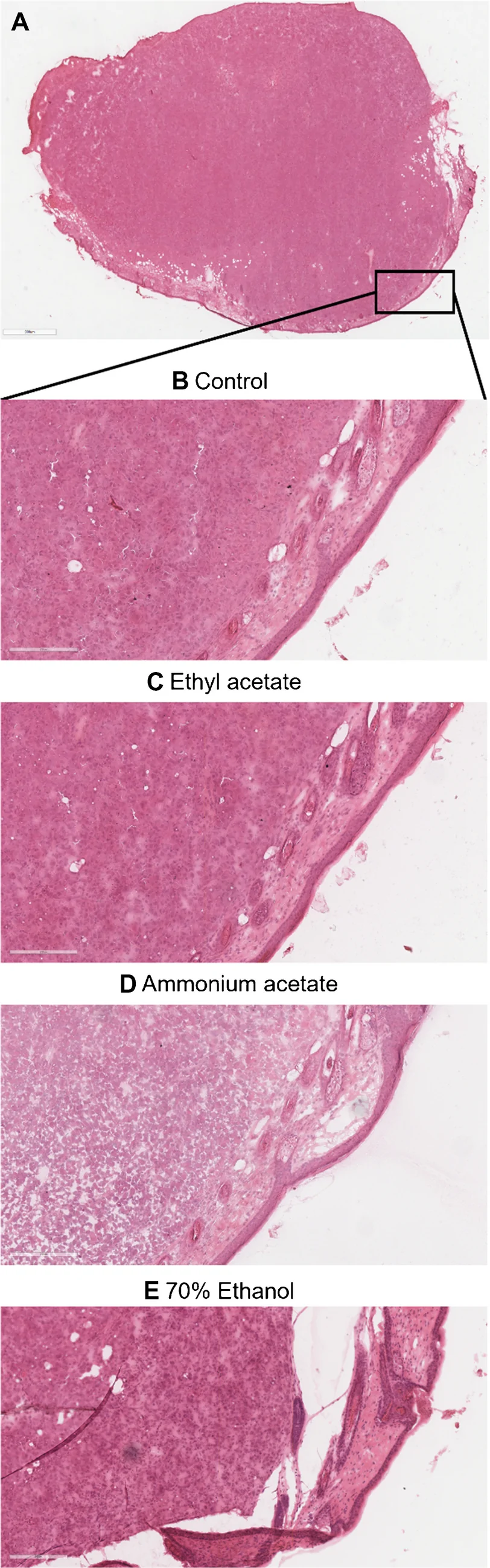
H&E-stained section of a PANC-1 xenograft tumour following different wash treatments. (**A**) Overview of the whole tumour with the area used for higher-magnification images marked. (**B**) High-magnification control section without wash. High-magnification sections after washing with (**C**) ethyl acetate, (**D**) ammonium acetate, and (**E**) ethanol (70% *v*/*v*)

MSI is often integrated into multimodal workflows where various staining protocols are applied post-MSI. In this context, it is vital that the selected wash treatments retain the initial tissue morphology. To assess tissue integrity after solvent treatment, tissues were stained with H&E. The resulting images showed varying structural integrity depending on the solvent treatment (Fig. 5). For EtOAc, the cell structure remained intact, with no separation or folding observed; furthermore, the colours remained comparable to those of the untreated control. In ammonium acetate-washed tissues, cells on the right side (the downward side during washing) showed signs of displacement in the corresponding H&E images. Overall, the cells are a lighter colour than the control cells, but their structure remains clearly visible, and histological quality is maintained. Tissues washed with 70% (*v*/*v*) ethanol also showed compromised structural integrity, including partial separation from the slide, poorly defined cellular morphology, and poor histological quality. Furthermore, ethanol washes led to severe drying, flaking, and partial separation from the glass slide during DESI-MSI analysis.

### Practical recommendations

**Fig. 6:**
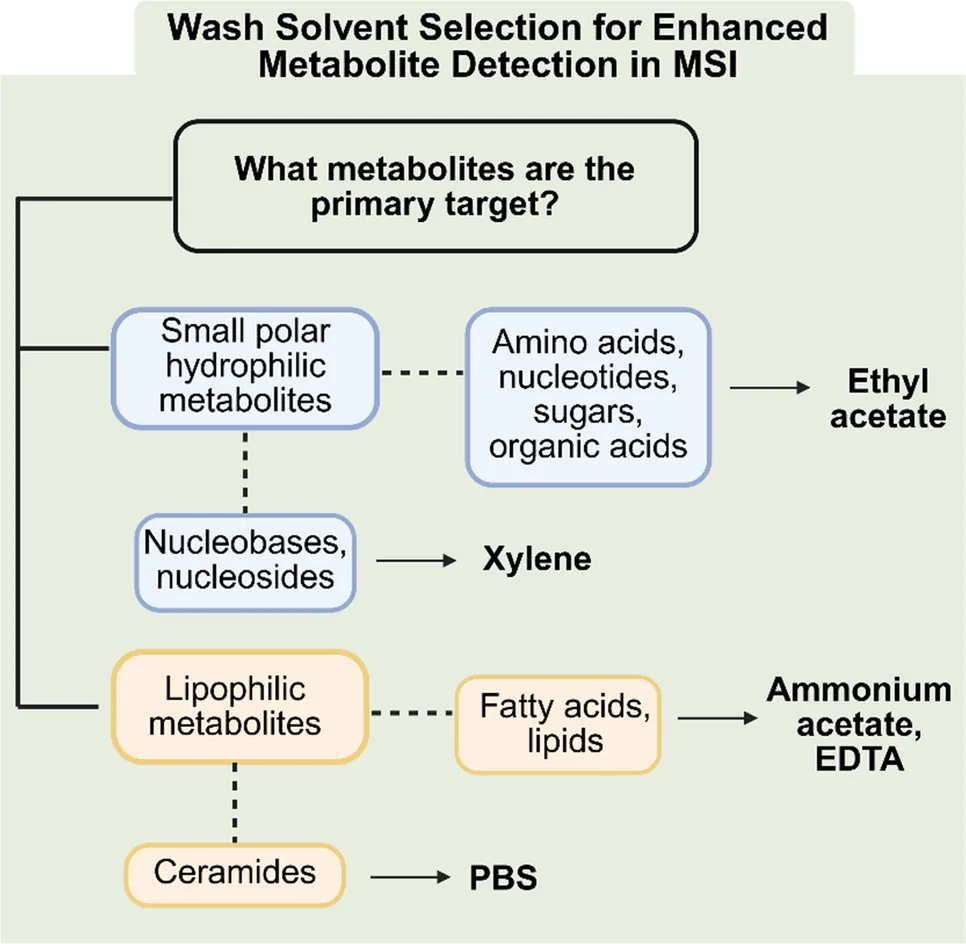
Decision-tree guide for selecting wash solvents to enhance metabolite detection in DESI-MSI on a metabolite class basis. Created in BioRender. Matic, B. (2026) https://BioRender.com/16jvm7y

For the detection of hydrophilic SMM, EtOAc is recommended, since it most consistently increased intensities across amino acids (all subclasses), nucleobases, nucleosides, nucleotides, sugars, and organic acids, with minimal delocalisation and no detectable morphological damage. However, EtOAc reduced the intensity of fatty acids and phospholipids due to its hydrophobic nature. EtOAc has recently been found to enhance the signal of the PET tracer UCB-J, which was ascribed to its ability to remove a broader spectrum of both polar and neutral lipids [22]. This study, however, did not assess chloroform as an alternative washing solvent. Our results show that chloroform was the most effective solvent for removing fatty acids and phospholipids; however, this did not automatically translate into improved detectability of smaller metabolites. SMMs were also largely reduced in intensity. Although chloroform has a slightly lower polarity index than ethyl acetate (4.1 vs 4.4), it has the ability to form (weak) hydrogen bonds, which facilitate its coordination with a broad range of organic molecules, making it an efficient solvent for extracting a wide range of compounds. The removal of most SMMs might be attributed to this ability [23]. Ethanol (70%, *v*/*v*) increased select metabolites strongly (e.g. adenine) but frequently caused metabolite delocalisation and tissue integrity issues (see Figs. 4 and 5), limiting its overall utility. MTBE and xylene slightly preserved or produced small increases for non-polar amino acids, sugars, and some organic acids, but had limited effects on polar metabolites and caused strong removal of fatty acids and phospholipids. In contrast, aqueous washes (PBS, 4% (*w*/*v*) formalin, ammonium acetate, EDTA) are recommended when enhanced detection of lipophilic metabolites is desired, as they increase the intensities of fatty acids and phospholipids while removing polar metabolites (amino acids, nucleic acids, sugars, organic acids). PBS uniquely enhanced ceramides, which, unlike the other lipid classes here, are detected as [M + Cl]^−^ adduct, whereas other washing interventions resulted in intensity losses for the assessed ceramide lipids. These findings are summarised in a wash solvent selection guide in Fig. 6.

## Conclusions

Wash steps are a promising tool to increase sensitivity, as using the right solvent system can increase intensity for all metabolites tested. As a systematic comparison of solvent systems for a wide range of analytes was lacking, we have performed a comparative study of 12 organic and aqueous wash solvents and their effects on various small molecule metabolites in tumour tissue. This resource is accessible as a public dataset on the Metaspace repository. It can serve as a tool for the MS imaging community to select a suitable wash solvent in a data-driven manner. Furthermore, it might serve as a useful basis to select suitable solvent compositions and modifiers in DESI-MSI to enhance analyte extraction and detection. Additionally, the impact of each solvent on tissue integrity was evaluated. To enable rapid, informed selection of appropriate wash solvents for individual target metabolites, a practical decision tree was provided.

The study presented here was performed using DESI-MSI in negative ion mode only; however, we expect the results to be largely transferable to other imaging modalities, such as MALDI or LA-REIMS, and positive polarity. Furthermore, the study presented here has been performed on tumour tissue only. While tumour is arguably the most widely used tissue in MSI studies, it is not unlikely that different effects will be observed across tissues, due to differences in chemical and mechanical properties that might affect solvent penetration and compound extraction.

## Supplementary Information

Below is the link to the electronic supplementary material.

Supplementary file1 (PDF 1.24 MB)

## Data Availability

Data are available under the Metaspace repository. Access links can be found in Supplementary Table S2.

## Declarations

### Source of biological material

Tumours were generated by subcutaneously injecting the human pancreatic cancer cell line PANC-1 into the flanks of 6-week-old female nude mice (Crl:NU(NCr)-*Foxn1*^*nu*^ Charles River Laboratories).

### Statement of animal welfare

All animal experiments were conducted in accordance with internationally recognised guidelines for animal welfare and the principles of Replacement, Reduction, and Refinement (the 3Rs). We minimised the number of animals used by using tumours derived from a vehicle control cohort, and our procedures were designed to minimise animal suffering.
