# Supplementary material for: Systematic assessment of different wash solvents for the analysis of small molecule metabolites using mass spectrometry imaging
Source: Anal Bioanal Chem. 2026 May 1;418(20):6737–47. doi: 10.1007/s00216-026-06534-x (PMC13619734; doi:10.1007/s00216-026-06534-x)
Supplement: Supplementary file 1 [file 216_2026_6534_MOESM1_ESM.pdf]

SUPPLEMENTARY MATERIAL

Systematic assessment of different wash solvents for the analysis of small molecule metabolites using mass spectrometry imaging

Authors and Affiliations

Barbara Matic[1], Lieby Zborovsky[1], Min Qiu[1], Katrin Frank[2], Kivanc Görgülü[2,3], Nicole Strittmatter[1]

[1] Department of Biosciences, School of Natural Sciences, Technical University of Munich, Garching b. München 85748, Germany

[2] Comprehensive Cancer Center München, Institute for Tumor Metabolism, Klinikum rechts der Isar, Technical University of Munich, School of Medicine and Health, Munich, Bavaria, Germany

[3] Institute of Metabolism and Cell Death, Helmholtz Zentrum München, Neuherberg, Germany

Corresponding author: Prof. Nicole Strittmatter, PhD, nicole.strittmatter@tum.de

Table S1. Wash solvents used in this study, their log K_{OW}s, [1] and polarity indices.[2]

Solvent	Log K_{OW}	Polarity index
Phosphate buffered saline (PBS)		10.2 (water)
Ethanol (70%)	-0.3	6.3
Acetone	-0.24	5.1
Chloroform	1.97	4.1
Methyl tert-butyl ether (MTBE)	0.94	2.5
Xylene (mix of isomers)	3.2	2.5
Dimethylsulfoxide (DMSO)	-1.35	7.2
EDTA		10.2 (water)
Acetonitrile	-0.34	5.8
4% Formalin	0.35	10.2 (water)
Ammonium acetate		10.2 (water)
Ethyl acetate	0.73	4.4

Table S2. Metaspace access links for different pairs of wash solvents. Left and right denote position to control (middle section) as shown in the image on Metaspace.

<table>
<tr><th>Wash left</th><th>Wash right</th><th>Metaspace dataset name</th><th>Metaspace access link</th></tr>
<tr><td>Formalin</td><td>Acetonitrile</td><td>Lower part: WashExp_ACN_Fotm_AmAc_EtOAc__80-900-150um_neg</td><td rowspan="6">https://metaspace2020.org/project/WashExp-2026</td></tr>
<tr><td>DMSO</td><td>EDTA</td><td>WashExp_EDTA_DMSO_80-900_150um_neg</td></tr>
<tr><td>EtOH</td><td>MTBE</td><td>WashExp_EtOH_MTBE_80-900_150um_neg</td></tr>
<tr><td>Xylene</td><td>Chloroform</td><td>Upper part: WashExp_Chl_Xyl_Acetone_PBS_80-900_150um_neg</td></tr>
<tr><td>PBS</td><td>Acetone</td><td>Lower part: WashExp_Chl_Xyl_Acetone_PBS_80-900_150um_neg</td></tr>
<tr><td>Ethyl acetate</td><td>Ammonium acetate</td><td>Upper part: WashExp_ACN_Fotm_AmAc_EtOAc__80-900-150um_neg</td></tr>
</table>

Table S3. Analytes of interest tested in this study.

Name	Chemical formula	*m/z* theor.	*m/z* exp.	Adduct	FDR	log K_{OW}	Molecular subclass
Alanine	$C_3H_7NO_2$	88.0404	88.0391	[M-H]-	20%	-2.74	Amino Acids
Valine	$C_5H_{11}NO_2$	116.0717	116.0705	[M-H]-	20%	-2.26	Amino Acids
(Iso-)Leucine	$C_6H_{13}NO_2$	130.0874	130.0862	[M-H]-	20%	-1.72	Amino Acids
Methionine	$C_5H_{11}NO_2S$	148.0438	148.0428	[M-H]-	50%	-1.87	Amino Acids
Proline	$C_5H_9NO_2$	114.0561	114.0549	[M-H]-	10%	-2.7	Amino Acids
Phenylalanine	$C_9H_{11}NO_2$	164.0717	164.0708	[M-H]-	20%	-1.38	Amino Acids
Tryptophan	$C_{11}H_{12}N_2O_2$	203.0826	203.0821	[M-H]-	20%	-1.06	Amino Acids
Serine	$C_3H_7NO_3$	104.0353	104.0341	[M-H]-	20%	-3.07	Amino Acids
Threonine	$C_4H_9NO_3$	118.0510	118.0498	[M-H]-	20%	-2.94	Amino Acids
Asparagine	$C_4H_8N_2O_3$	131.0462	131.0451	[M-H]-	20%	-3.4	Amino Acids
Glutamine	$C_5H_{10}N_2O_3$	145.0619	145.0608	[M-H]-	10%	-3.3	Amino Acids
Tyrosine	$C_9H_{11}NO_3$	180.0666	180.0659	[M-H]-	50%	-2.26	Amino Acids
Arginine	$C_6H_{14}N_4O_2$	173.1044	173.1036	[M-H]-	20%	-4.2	Amino Acids
Lysine	$C_6H_{14}N_2O_2$	145.0983	145.0972	[M-H]-	50%	-3.05	Amino Acids
Histidine	$C_6H_9N_3O_2$	154.0622	154.0612	[M-H]-	10%	-3.32	Amino Acids
Aspartate	$C_4H_7NO_4$	132.0302	132.0291	[M-H]-	10%	-3.5	Amino Acids
Glutamate	$C_5H_9NO_4$	146.0459	146.0449	[M-H]-	5%	-3.5	Amino Acids
Adenine	$C_5H_5N_5$	134.0472	134.0461	[M-H]-	20%	-0.38	Nucleic Acid Components
Uracil	$C_4H_4N_2O_2$	111.0200	111.0188	[M-H]-	10%	-1.2	Nucleic Acid Components
Guanine	$C_5H_5N_5O$	150.0421	150.0411	[M-H]-	50%	-0.92	Nucleic Acid Components
Thymine	$C_5H_6N_2O_2$	125.0357	125.0345	[M-H]-	50%	-0.99	Nucleic Acid Components
Xanthine	$C_5H_4N_4O_2$	151.0262	151.0252	[M-H]-	10%	-0.65	Nucleic Acid Components
Hypoxanthine	$C_5H_4N_4O$	135.0312	135.0303	[M-H]-	10%	-0.55	Nucleic Acid Components
Adenosine	$C_{10}H_{13}N_5O_4$	302.0662	302.0664	[M+Cl]-	5%	-1.2	Nucleic Acid Components
Guanosine	$C_{10}H_{13}N_5O_5$	282.0844	282.0845	[M-H]-	10%	-2.1	Nucleic Acid Components
Uridine	$C_9H_{12}N_2O_6$	243.0623	243.0622	[M-H]-	5%	-1.8	Nucleic Acid Components
AMP	$C_{10}H_{14}N_5O_7P$	346.0558	346.0561	[M-H]-	5%	-3.1	Nucleic Acid Components
UMP	$C_9H_{13}N_2O_9P$	323.0286	323.0289	[M-H]-	5%	-1.8	Nucleic Acid Components
GMP	$C_{10}H_{14}N_5O_8P$	362.0507	362.0512	[M-H]-	5%	-3.3	Nucleic Acid Components
CMP	$C_9H_{14}N_3O_8P$	322.0446	322.0448	[M-H]-	5%	-2	Nucleic Acid Components
Hexose	$C_6H_{12}O_6$	215.0328	215.0324	[M+Cl]-	5%	-2.6	Carbohydrates
Pentose	$C_5H_{10}O_5$	149.0455	149.0446	[M-H]-	50%	-2.4	Carbohydrates
Deoxy-hexose	$C_6H_{12}O_5$	199.0379	199.0373	[M+Cl]-	50%	-2	Carbohydrates

Di-hexose	$C_{12}H_{22}O_{11}$	377.0856	377.086	[M+Cl]-	20%	-3	Carbohydrates
Glutathione	$C_{10}H_{17}N_3O_6S$	306.0765	306.0768	[M-H]-	5%	-2.7	Organic acids
Uric acid	$C_5H_4N_4O_3$	167.0211	167.0202	[M-H]-	20%	-2.17	Organic acids
Vitamin C (Ascorbic acid)	$C_6H_8O_6$	175.0248	175.024	[M-H]-	5%	-1.6	Organic acids
Citric acid	$C_6H_8O_7$	191.0197	191.0191	[M-H]-	10%	-1.3	Organic acids
Vitamin B5 (Panthothenic acid)	$C_9H_{17}NO_5$	218.1034	218.1031	[M-H]-	5%	-1.1	Organic acids
Malic acid	$C_4H_6O_5$	133.0142	133.0131	[M-H]-	10%	-0.87	Organic acids
α-Ketoglutaric acid	$C_5H_6O_5$	145.0142	145.0132	[M-H]-	50%	-0.6	Organic acids
Succinic acid	$C_4H_6O_4$	117.0193	117.0182	[M-H]-	5%	-0.53	Organic acids
Lactic acid	$C_3H_6O_3$	89.0244	89.0231	[M-H]-	5%	-0.47	Organic acids
Aconitic acid	$C_6H_6O_6$	173.0092	173.0085	[M-H]-	10%	-0.41	Organic acids
Pyruvic acid	$C_3H_4O_3$	87.0088	87.0075	[M-H]-	20%	-0.38	Organic acids
Itaconic acid	$C_5H_6O_4$	129.0193	129.0182	[M-H]-	20%	0.053	Organic acids
Fumaric acid	$C_4H_4O_4$	115.0037	115.0025	[M-H]-	20%	0.21	Organic acids
C16:1	$C_{16}H_{30}O_2$	253.2173	253.2174	[M-H]-	5%	5.33	Fatty acids
C18:0	$C_{18}H_{36}O_2$	283.2643	283.2644	[M-H]-	5%	6.33	Fatty acids
C18:2	$C_{18}H_{32}O_2$	279.2330	279.2331	[M-H]-	5%	5.88	Fatty acids
C20:4	$C_{20}H_{32}O_2$	303.2330	303.2332	[M-H]-	5%	6.22	Fatty acids
C22:6	$C_{22}H_{32}O_2$	327.2330	327.2333	[M-H]-	5%	6.55	Fatty acids
C24:4	$C_{24}H_{40}O_2$	359.2956	359.296	[M-H]-	5%	7.78	Fatty acids
Cer(34:1;O2)	$C_{34}H_{67}NO_3$	572.4815	572.4823	[M+Cl]-	5%	10.42	Lipids
Cer(32:1;O2)	$C_{32}H_{63}NO_3$	544.4502	544.4509	[M+Cl]-	20%	9.64	Lipids
Cer(36:1;O2)	$C_{36}H_{71}NO_3$	600.5128	600.5139	[M+Cl]-	20%	11.2	Lipids
PI(36:4)	$C_{45}H_{79}O_{13}P$	857.5186	857.5196	[M-H]-	5%	11.13	Lipids
PI(34:1)	$C_{43}H_{81}O_{13}P$	835.5342	835.5345	[M-H]-	20%	11.03	Lipids
PI(36:2)	$C_{45}H_{83}O_{13}P$	861.5499	861.5511	[M-H]-	10%	11.58	Lipids
PI(36:1)	$C_{45}H_{85}O_{13}P$	863.5655	863.5655	[M-H]-	10%	11.81	Lipids
PA(32:0)	$C_{35}H_{69}O_8P$	647.4657	647.4667	[M-H]-	10%	11.71	Lipids
PA(34:2)	$C_{37}H_{69}O_8P$	671.4657	671.4671	[M-H]-	10%	12.04	Lipids
PA(36:2)	$C_{39}H_{73}O_8P$	699.4970	699.4981	[M-H]-	5%	12.82	Lipids
PG(36:1)	$C_{42}H_{81}O_{10}P$	775.5495	775.5503	[M-H]-	10%	13.11	Lipids
PG(36:2)	$C_{42}H_{79}O_{10}P$	773.5338	773.5344	[M-H]-	10%	12.89	Lipids
PS(40:5)	$C_{46}H_{80}NO_{10}P$	836.5447	836.5446	[M-H]-	5%	13.55	Lipids
PS(36:1)	$C_{42}H_{80}NO_{10}P$	788.5447	788.5469	[M-H]-	5%	12.88	Lipids
PS(38:4)	$C_{44}H_{78}NO_{10}P$	810.5291	810.5305	[M-H]-	5%	12.99	Lipids
LPS(20:4)	$C_{26}H_{44}NO_9P$	544.2681	544.2688	[M-H]-	20%	6.18	Lipids
PE(O-36:5)	$C_{41}H_{74}NO_7P$	722.5130	722.5143	[M-H]-	5%	13.29	Lipids
LPE(20:4)	$C_{25}H_{44}NO_7P$	500.2783	500.279	[M-H]-	5%	6.72	Lipids

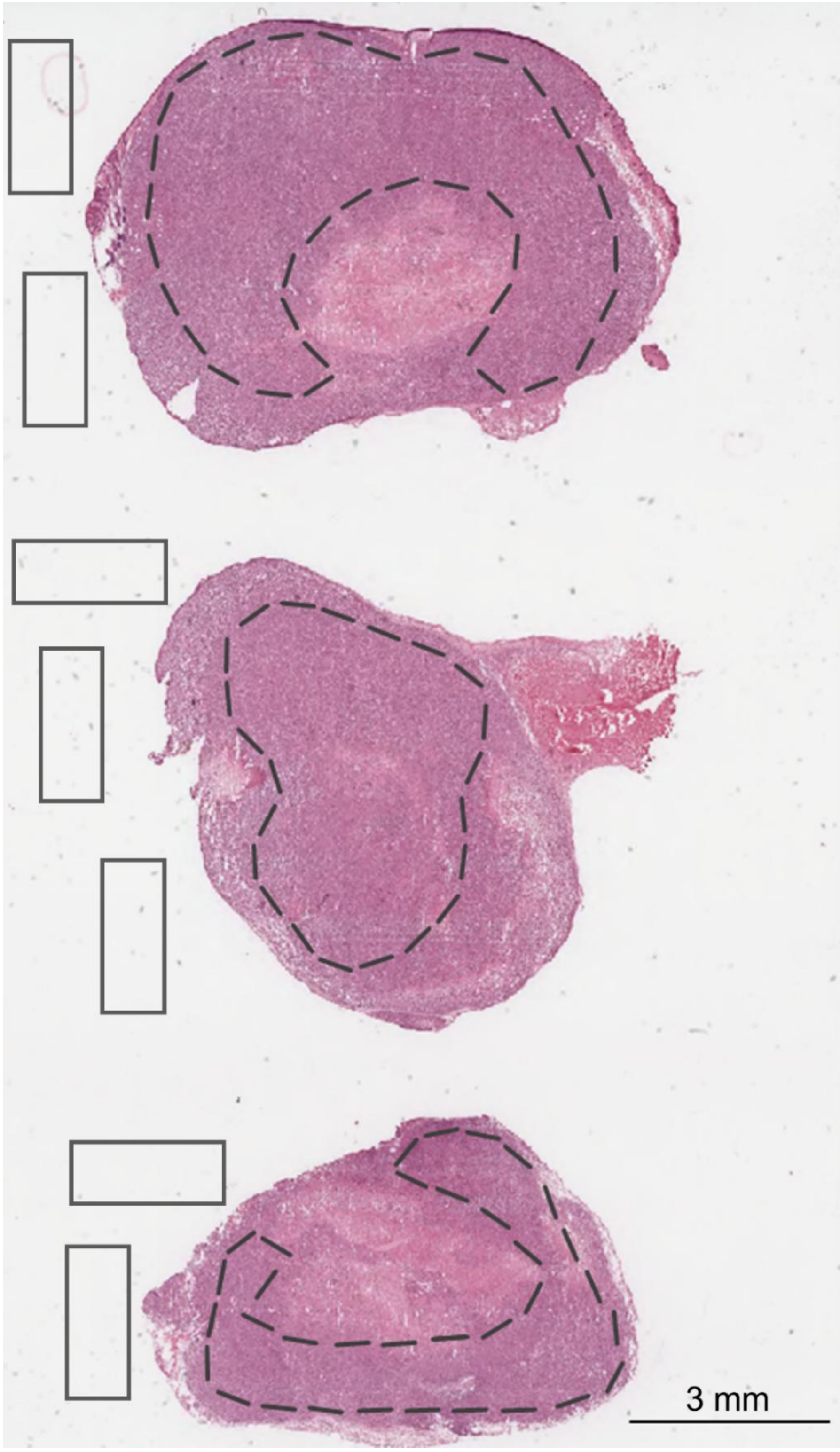

Figure S1. H&E-stained section of a PANC-1 xenograft tumour with the tumour region of interest (ROI) outlined by a dashed line. On the side, the areas used to calculate the degree of delocalisation are shown; the downward-facing side was used for analysis.

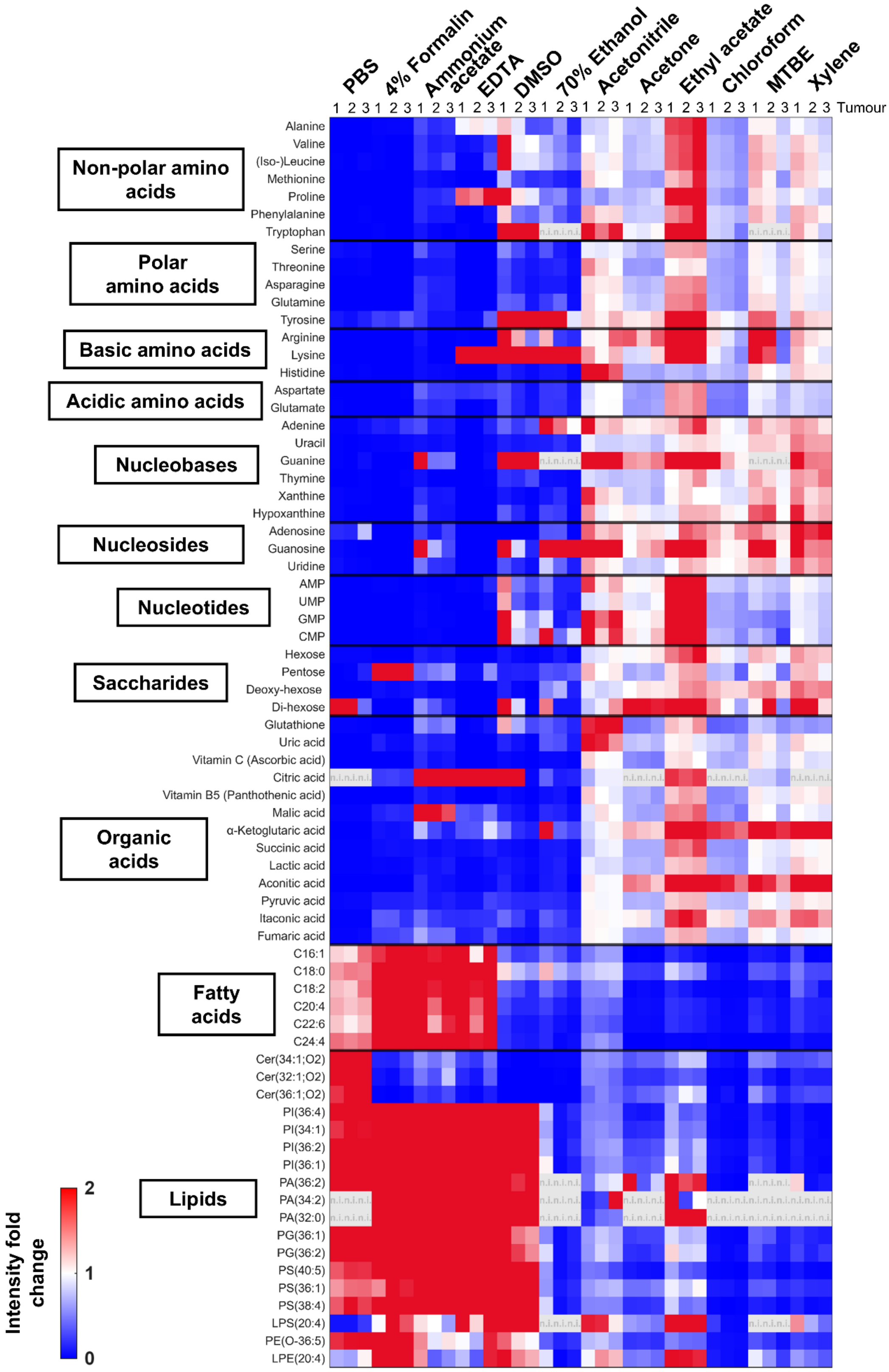

Figure S2. Intensity fold change in treated vs. control tumours across metabolite classes. Solvents are arranged in order of decreasing polarity (left to right). 0 = complete removal, 1 = no change, ≥2 = ≥two-fold increase. n.i., not detected.

Additional discussion on amino acid intensity enhancements:

Amino Acids. Among non-polar amino acids (alanine, valine, (iso-)leucine, methionine, proline, phenylalanine, and tryptophan), ethyl acetate washing produced the greatest increases in intensity, with tryptophan and proline exhibiting the largest enhancements. MTBE and xylene washes resulted in slight increases in signal intensity, whereas acetonitrile selectively enhanced the signals of larger non-polar amino acids (methionine, phenylalanine, and tryptophan). DMSO showed heterogeneous effects, increasing tryptophan, proline, valine, and (iso-)leucine while reducing alanine, methionine, and phenylalanine, while EDTA resulted in a selective increase in proline and alanine.

Polar, yet uncharged, amino acids (serine, threonine, asparagine, glutamine, and tyrosine) demonstrated increased intensities following ethyl acetate washing, with acetonitrile also producing modest enhancements. Tyrosine was uniquely responsive to both non-polar and polar organic solvents, including DMSO and ethanol, whereas the remaining polar amino acids did not show significant increases with other organic solvent treatments. Aqueous washes resulted in near-complete removal of polar amino acids due to their strong hydrophilic characteristics.

Basic amino acids (arginine and lysine) showed enhanced intensities following ethyl acetate washing and were slightly increased after all organic solvent washes except chloroform. Histidine showed a slight decrease in intensity after most organic solvent washes, except for acetonitrile, which significantly increased its relative intensity. While lysine demonstrated substantial intensity increases, organic EDTA, DMSO, and ethanol washes resulted in metabolite delocalisation.

Acidic amino acids (aspartate and glutamate) were only enhanced by ethyl acetate washing, whereas other organic solvents did not significantly increase their intensities. Aqueous washes resulted in the removal of acidic amino acids.

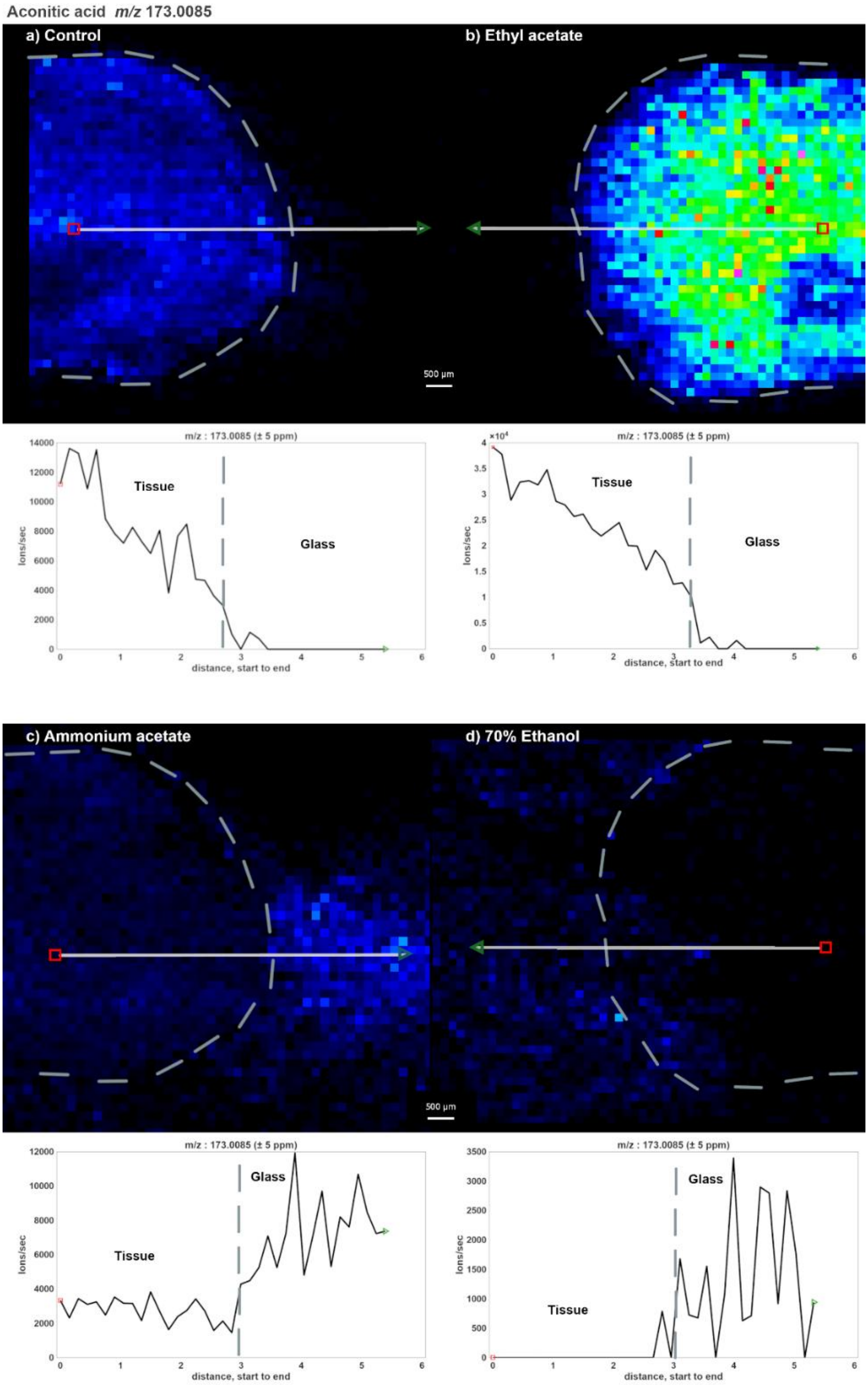

Figure S3. Spatial distribution of aconitic acid (m/z 173.0085 ± 5 ppm) following different solvent treatments. Spatial resolution is 150 µm. Representative ion images of tissue sections are shown for (a) unwashed control, (b) ethyl acetate (EtOAc), (c) ammonium acetate, and (d) 70% (v/v) ethanol. Dashed lines show the tissue boundaries. Corresponding line plots depict intensity from the tissue to the glass, following the direction indicated by the arrows in the images.

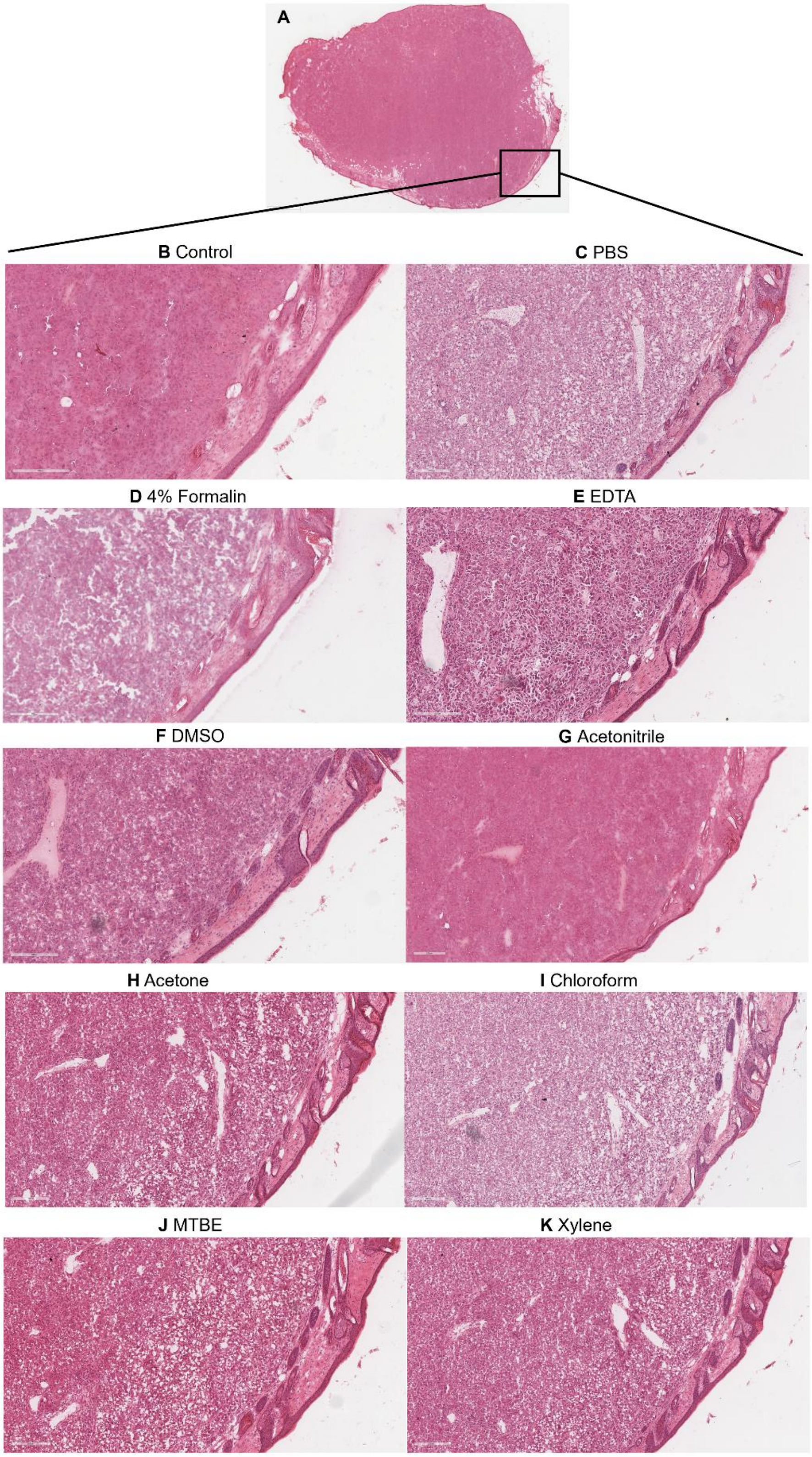

Figure S4. H&E-stained section of a PANC-1 xenograft tumour following different wash treatments. a) Low-magnification overview of the whole tumour with the area used for higher-magnification images marked. b) High-magnification control section without wash. High-magnification sections after washing with c) PBS, d) 4% formalin, e) EDTA, f) DMSO, g) acetonitrile, h) acetone, i) chloroform, j) MTBE, and k) xylene.

References

1. Sangster J. Octanol-Water Partition Coefficients of Simple Organic Compounds. Journal of Physical and Chemical Reference Data. 1989; https://doi.org/10.1063/1.555833
2. Snyder LR. Classification off the Solvent Properties of Common Liquids. Journal of Chromatographic Science. 1978; https://doi.org/10.1093/chromsci/16.6.223
